# Fatty acid synthesis in *Escherichia coli* and its applications towards the production of fatty acid based biofuels

**DOI:** 10.1186/1754-6834-7-7

**Published:** 2014-01-09

**Authors:** Helge Jans Janßen, Alexander Steinbüchel

**Affiliations:** 1 Institut für Molekulare Mikrobiologie und Biotechnologie, Westfälische Wilhelms-Universität Münster, Corrensstrasse 3, D-48149, Münster, Germany; 2 Environmental Sciences Department, King Abdulaziz University, Jeddah, Saudi Arabia

**Keywords:** Biofuels, *Escherichia coli*, Fatty acid biosynthesis, Regulation

## Abstract

The idea of renewable and regenerative resources has inspired research for more than a hundred years. Ideally, the only spent energy will replenish itself, like plant material, sunlight, thermal energy or wind. Biodiesel or ethanol are examples, since their production relies mainly on plant material. However, it has become apparent that crop derived biofuels will not be sufficient to satisfy future energy demands. Thus, especially in the last decade a lot of research has focused on the production of next generation biofuels. A major subject of these investigations has been the microbial fatty acid biosynthesis with the aim to produce fatty acids or derivatives for substitution of diesel. As an industrially important organism and with the best studied microbial fatty acid biosynthesis, *Escherichia coli* has been chosen as producer in many of these studies and several reviews have been published in the fields of *E. coli* fatty acid biosynthesis or biofuels. However, most reviews discuss only one of these topics in detail, despite the fact, that a profound understanding of the involved enzymes and their regulation is necessary for efficient genetic engineering of the entire pathway. The first part of this review aims at summarizing the knowledge about fatty acid biosynthesis of *E. coli* and its regulation, and it provides the connection towards the production of fatty acids and related biofuels. The second part gives an overview about the achievements by genetic engineering of the fatty acid biosynthesis towards the production of next generation biofuels. Finally, the actual importance and potential of fatty acid-based biofuels will be discussed.

## Introduction

During the recent decades it has become evident that the world’s fossil fuel reserves are decreasing and will be most probably depleted rather soon. However, until 2016 the global demand for crude oil will increase by more than 1 million barrels per day [1], and also for the time after 2018 no dramatic change in energy need is expected. As a result governments, companies and scientists work on the development of sustainable ways to produce energy. Concerning biofuels there are actually two major products of great commercial importance: ethanol and biodiesel (fatty acid alkyl ester, FAAE), which currently account for roughly 90% of the biofuel market [2,3].

The main producers of bioethanol are the USA and especially Brazil, where the gasoline must be blended with at least 25% ethanol [4,5]. The production of ethanol is based on fermentation of *Saccharomyces cerevisiae* and the most often used substrates are carbohydrates obtained from sugarcane, corn, wheat, sugar beet and some other plants [6]. In comparison to petrol, bioethanol and bioethanol blends have a higher cetane number, but a roughly 30% lower energy density [7]. Another problem is the hygroscopicity of ethanol, which makes storage and transportation challenging [8]. In theory, bioethanol can be carbon-neutral as plant material is used for its production. But due to the kind of fermentation process, forest clearance, intensive use of fertilizers and the energy consumption during distillation, the overall emissions of CO_2_ exceed its consumption. In addition, other pollutants such as mono-nitrogen oxides (NO_x_) or carbon monoxide (CO) are produced, which result in an environmental impact that might even be worse than from the use of fossil fuels [9,10].

Biodiesel is produced by the transesterification of mostly plant-derived triacylglycerols (TAG), yielding glycerol and FAAEs. Methanol is being used as alcohol moiety, due to its low price [11]. In contrast to ethanol, biodiesel has very similar properties to petrol and can therefore be used in the same engines and distributed through the same infrastructure. Like ethanol, it has some environmentally friendly aspects because it is degradable, its biosynthesis consumes carbon dioxide (CO_2_), and it has low sulfur content when compared to crude oils. However, most studies conclude that the overall environmental impact of biodiesel is also negative [12], due to the use of fertilizers for growing the oil plants and the transesterification process, which is energy consuming and relies on the use of toxic methanol [11].

**Figure 1:**
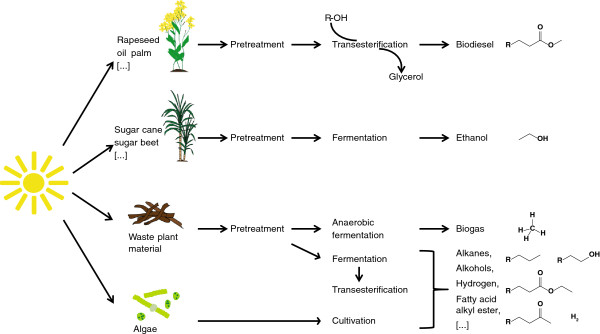
Overview of the production of first- and second-generation biofuels.

Another drawback of currently used biofuels is that to date all economically feasible processes are based on the utilization of cereal crops (for example, wheat, and maize), oil crops (for example rape, palm oil and soya) or sugar crops (for example, sugar beet and sugar cane) [6,13]. In the last years this has led to intensive political debate concerning the social and environmental consequences of the use of food and agricultural land for biofuel production [9,14-16]. Owing to these drawbacks one speaks of first-generation biofuels in contrast to second-generation biofuels (Figure 1) which rely on the use of lignocellulose or other feedstocks that do not directly compete with food, and reduce the need for agricultural land [13].

As an alternative to the use of plant-derived TAGs, the microbial production of free fatty acids (FFA) or FFA-derived biofuels offers a great potential due to short production times and very low land-use. To reduce the competition with food, the use of cellulose, lignin, hemicellulose, CO_2_ or other non-food carbon sources needs further optimization, although many strategies have already been established [17-19] and their suitability for biofuel production has been shown [20-23]. Furthermore, direct microbial production of FAAE has also been published, which makes the subsequent transesterification unnecessary and thus saves energy costs and reduces the use of methanol [24-26]. Some recent investigations lead to the production of fatty acid-derived alkanes, alcohols, methylketones or 3-hydroxyalkanoates. All mentioned compounds are suitable as diesel replacement. The production of TAG in *Escherichia coli* (*E. coli*) may in future become an alternative for TAG production by native producers (like species of the genera *Rhodococcus*, *Mycobacterium* or *Streptomyces*) that mostly exhibit a rather slow growth rate and are not as easy to genetically modify as *E. coli*.

To date, promising results for microbial production of free fatty acids and derived products have been obtained mainly by metabolic engineering, but for the production of large amounts of cheap biofuels much effort still needs to be undertaken. For this a detailed knowledge about the participating enzymes and their regulation is crucial. The objective of this review is to focus on the biosynthesis of fatty acids in the fast growing and industrially important microorganism *E. coli*. Especially, we will sum up possibilities to genetically modify this bacterium towards an overproduction of fatty acids or fatty acid-derived biofuels.

### Fatty acid biosynthesis

Synthesis of fatty acids is one of the most ubiquitous pathways in organisms. In eukaryotic and prokaryotic cells fatty acids are precursors for a variety of important building blocks such as phospholipids, sphingolipids, sterols, as secondary metabolites and signaling molecules, or they are attached to proteins. By changing the grade of saturation of the phospholipids in cellular membranes, their fluidity can be altered, which makes an adaption to temperature changes possible. Because the degradation of fatty acids yields a high amount of ATP and reducing equivalents, they also represent a suitable storage compound for energy and carbon. Especially in multicellular organisms, but also in unicellular eukaryotes and prokaryotes, fatty acids are stored as TAG or wax esters, whereas the storage of hydroxyfatty acids as polyhydroxyalkanoates is limited to bacterial species. In *Archaea*, fatty acids play a minor role, owing to their differing membrane, which mainly consists of fatty alcohol-glycerol diethers instead of fatty acid-glycerol diesters. Nevertheless, fatty acid biosynthesis is also performed by *Archaea*, and the products can be used to acylate membrane proteins [27,28].

Despite the early development of fatty acid biosynthesis during evolution of life but due to its importance, the pathway is highly conserved within the kingdoms of life. At the first step, malonyl-CoA is formed by carboxylation of acetyl-CoA with hydrogencarbonate, by the expense of ATP. Coenzyme A is then exchanged by the acyl carrier protein (ACP) resulting in malonyl-ACP. The ACP prevents the growing fatty acid chain from degradation and from being used for anabolic reactions. With malonyl-ACP, the first turn of the fatty acid biosynthesis cycle starts by an initial condensation of malonyl-ACP with acetyl-CoA, yielding hydrogencarbonate, free coenzyme A and acetoacetyl-ACP. The latter is then reduced to 3-hydroxybutyryl-ACP, dehydrated to 2-butenoyl-ACP and further reduced to butyryl-ACP. Butyryl-ACP enters the next turn of the cycle again by a condensation with malonyl-ACP. Fatty acid synthesis stops when a certain chain length is reached, and the acyl-ACP is used for membrane synthesis. As both reduction steps require two reduction equivalents, derived from nicotinamide adenine dinucleotide (NADPH), the following equation for the elongation of a fatty acid by a two-carbon unit applies:

$$\begin{array}{l} C_{n}H_{2n-1}O_{2}-\mathrm{ACP}+C_{3}O_{3}H_{3}-\mathrm{ACP}+\mathrm{ATP}\\ \;+2\;\mathrm{NAD}(P)H+2H^{+} \end{array}$$

$$\begin{array}{l} \to C_{n+2}H_{2n+3}O_{2}-\mathrm{ACP}+{{\mathrm{HCO}}_{3}}^{–}+\mathrm{ADP}+P_{i}\\ \;+2\;\mathrm{NAD}{\left(P\right)}^{+} \end{array}$$

Despite high similarities in the general pathway, different enzymes and different genetic organizations have evolved. In animals and fungi, the type-I fatty acid synthase caries out all steps of fatty acid biosynthesis as one multifunctional protein complex. This type is further divided into the fungal typeIa, in which the fatty acid synthase is encoded by two genes and is assembled to a α_6_β_6_ heterododecamer of about 2.6 MDa. Fatty acid synthase typeIb is found in animals. Here, all required proteins are encoded by a single gene, and the translated peptide chains form an α_2_ homodimer of about 540 kDa [29,30].

Type-II fatty acid synthase is predominant in prokaryotes as well as in the plastids of plants, in which *de novo* synthesis of plant fatty acids takes place [31-34]. An exception are Gram-positive, mycolic acid-producing bacteria, which contain a type-I fatty acid synthase as one polypeptide chain [35-37] and additionally a type-II fatty acid synthase, which is only involved in the elongation of fatty acids with medium chain length but cannot start *de novo* fatty acid biosynthesis [38,39]. The main difference of FAS type-II is that it consists of a set of enzymes that are not organized as one single gene or operon. In any case, in many bacteria such as *E. coli*, a number of the genes are organized in the *fab* cluster. The bacterial acetyl-CoA carboxylase represents an example for a bacterial enzyme complex that is involved in fatty acid biosynthesis.

Studies of the fatty acid synthase of *Archaea* are rare, because the *de novo* synthesis of fatty acids seems not to be comparably important as in other organisms. The use of fatty acids as anchor molecules for membrane proteins has been proven [28], and additionally they have been found as parts of phospholipids [40] and as free fatty acids [41,42]. Due to similarities with the bacterial fatty acid profile [43,44] and within the inhibition pattern at high salt-concentration of archaeal and bacterial FAS, as well as in the isolation procedure, it has been suggested that *Archaea* contain a type-II FAS [45].

## The *E. coli* type-II FAS enzymes

**Figure 2:**
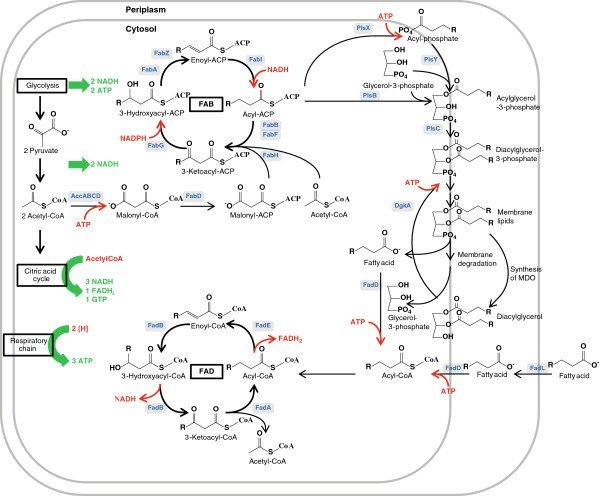
**Biosynthesis and degradation of fatty acids and membrane lipids.** ATP and reduction equivalents are colored red if consumed and green if gained. Enzymes are colored blue. FAB, fatty acid biosynthesis; FAD, fatty acid degradation.

This section deals with the set of enzymes that perform the fatty acid biosynthesis of *E. coli*. Transcriptional and biochemical regulation is emphasized, and studies concerning overexpression or deletion of the respective genes are discussed with special interest in the impact on overproduction of fatty acids. The enzymes and regulation of the membrane synthesis and fatty acid degradation are also of great interest, as they represent the main competing pathways with a FFA overproduction. An overview of the involved pathways is shown in Figure 2.

### AccABCD: acetyl-CoA carboxylase

Acetyl-CoA carboxylase represents the starting enzyme and directs acetyl-CoA towards *de*-*novo* fatty acid biosynthesis and chain elongation. In *E. coli*, the four subunits form a very unstable complex that could be purified as two subcomplexes: 1) the biotin carboxylase-biotin carboxyl carrier protein (BC-BCCP), which is a homodimer of AccC (biotin carboxylase), interacting with four molecules of AccB (biotin carboxyl carrier protein) [46], and 2) the carboxyl transferase, which is a heterotetramer, consisting of two subunits of AccA and two subunits of AccD [47]. The reaction can be divided into two half-reactions with (i) the carboxylation of biotin, by the expense of ATP and with Mg^2+^-ions as cofactor and (ii) the subsequent transfer of the carboxyl group to acetyl-CoA, yielding malonyl-CoA [47].

Transcription of *accABCD* is strictly coordinated and regulated, as the subunits have to be synthesized in equimolar amounts. In addition, the carboxylation of acetyl-CoA is driven by cleavage of ATP and thus consumes energy. The genes *accB* and *accC* build one mRNA, which serves as a template for the translation of both AccB and AccC [48]. Their transcription, which positively correlates with the growth rate, is further autoregulated by the N-terminal domain of AccB [49,50]. Overexpression of *accB* inhibits the transcription of *accB* and *accC*, whereas in an *accB*-deletion mutant the transcription of the *accBC* operon is not altered [50]. Additionally, the excess of AccB deregulates the biotin synthetic operon and thus, the cells are stressed due to the strong biotin-synthesis [51,52]. Enhanced production of AccC also affects the biotin operon, as AccC forms complexes with AccB, which is more efficiently biotinylated in its unbound form. If AccC is more abundant, almost no biotin is attached to AccB, and transcription of the biotin operon is shut down by BirA, which has a dual function as biotin protein ligase and as repressor for the *bio*-operon [51]. The resulting reduction of fatty acid biosynthesis by AccB or AccC overexpression however, is not seen in a strain with overexpression of both proteins in equimolar amounts [53].

In contrast to *accB* and *accC*, the genes coding for the carboxyl transferase are not part of an operon, and no transcriptional regulation has been found by sequence analysis [49]. Instead, the translation of the respective mRNAs is controlled by the *β*-subunit (AccD) of the mature carboxyl transferase [54]. This subunit forms a zinc-finger motif, which binds to *accA* and *accD* mRNA, but is also required for the catalytic activity [55]. Since the binding of mRNA is preferred in comparison to the binding of acetyl-CoA, high levels of acetyl-CoA (as in growing cells) are required to resolve the complex of carboxyl transferase and its mRNA and thus, to promote both, synthesis of malonyl-CoA and translation of AccA and AccD [54]. As the zinc-finger motif is found in *E. coli* and *Staphylococcus aureus* AccD [56], as well as in the chloroplast-encoded *β*-subunit of the carboxyltransferase of pea, tobacco, rice, liverwort [57,58] and wheat but not in type-I fatty acid synthase, this leads to the assumption that this type of regulation might be common in type-II fatty acid synthases [56].

For the *E. coli* acetyl-CoA carboxylase it has been shown that the enzyme activity is inhibited by acyl-ACP with chain lengths of C6 to C20. Thus, an accumulation of fatty acids that are not used for membrane lipid synthesis is prevented [59].

Overexpression of different combinations of the four subunits of the acetyl-CoA carboxylase in equimolar amounts has been extensively studied by Davis *et al*. [60]. The normally very weak enzyme activity in cellular crude extracts could be enhanced 50-fold. Interestingly the overexpression of *accBCD* led to an 11-times enhanced activity, whereas all tested combinations of one or two subunits did not result in enhanced activity. Comparing this with the more recent results for the translational regulation of AccA and AccD [54], it seems reasonable that a higher copy-number of the mRNA of AccA or AccD, or both, lead to an overall higher level of translation of *accA* and *accD* mRNA. Translation will be higher than in the wild-type until equilibrium between the copy number of AccAD and the respective mRNAs is reached.

However, overproduction of AccABCD was found to result only in enhanced production of free fatty acids if a cytosolic form of thioesterase I (‘*tesA*) was coexpressed and thus a metabolic sink for fatty acids was provided. Nearly the same amount of free fatty acids was achieved upon coexpression of *accBCD* and ‘*tesA*, and the expression of ‘*tesA* alone still resulted in high amounts of free fatty acids. Additional coexpression of *birA*, to attach the biotin cofactor to AccB, did not enhance the enzyme activity significantly; therefore, biotin availability should not be a limiting factor [60]. In the study of Zha *et al*. [61], the level of malonyl-CoA in *E. coli* was increased 15-fold. They overexpressed the acetyl-CoA carboxylase, and additionally improved acetate assimilation and deleted the formation of ethanol and acetate.

### FabD: malonyl-CoA:ACP transacylase

Malonyl-CoA:ACP transacylase catalyzes the transfer of the malonyl-moiety to ACP, which directs it towards fatty acid neogenesis and fatty acid chain elongation. Kinetic studies have shown that the *E. coli* FabD cannot use acetyl-CoA as substrate but the latter shows a weak competitive inhibition of the reaction. Also the binding of ACP is inhibited by high CoA-SH concentrations. It was further shown that in presence of equal concentrations of substrates and products the formation of malonyl-CoA is favored over the production of malonyl-ACP [62].

Deletion of *fab*D has been shown to be lethal [63,64], and overexpression of *fab*D in *E. coli* leads to a slightly altered fatty acid composition. The proportion of palmitoleic acid was shown to decrease, whereas the proportion of *cis*-vaccenic acid increases [65]. The authors suggest that the different composition is caused by an enhanced malonyl-ACP pool and thus, enhanced activity of the 3-ketoacyl-ACP synthase II (FabF), which is the enzyme responsible for chain elongation of C16:1 to C18:1 [66]. Overexpression of the *E. coli fabD*, together with ‘*tesA*, increases the amount of free fatty acids by about 11% when compared to overexpression of the thioesterase gene alone [67]. The *fab*D gene is transcribed as part of the *fab*-cluster, which is described in more detail in the regulation section.

### FabB, FabF and FabH: 3-ketoacyl-ACP synthase I, II and III

The 3-ketoacyl-ACP synthase catalyzes the formation of 3-ketoacyl-ACP by condensation of fatty acyl-ACP with malonyl-ACP. In the case of FabH, the substrates are malonyl-ACP and acetyl-CoA, initiating the first cycle of chain elongation during fatty acid biosynthesis. Subsequent elongation steps are performed by FabF and FabB.

The activity of FabH with propionyl-CoA is as good as with acetyl-CoA, leading to the formation of fatty acids with an uneven number of carbon atoms. The activity with butyryl-CoA is much lower, and hexanoyl-CoA is no natural substrate of FabH [68]. The *E. coli* FabH exhibits no activity with branched-chain acyl-CoA esters, but heterologous expression of the *Bacillus subtilis fabH* gene leads to the formation of branched-chain fatty acids in *E. coli*[69].

The activities of FabF and FabB differ only in the catalysis of some reactions. Both show activity with C6 to C14 saturated fatty acyl-ACP esters; however, C14:0 is a weak substrate of both enzymes and only FabF exhibits low activity with C16:0 [70]. However, the condensation reaction with butyryl-ACP was not tested [70], but as activity of both enzymes with acetyl-ACP was found [71] it seems likely that butyryl-ACP is also a suitable substrate.

In the synthesis of unsaturated fatty acids, FabB catalyzes the condensation of *cis*-3-decenoyl-ACP (formed by the FabA catalyzed reaction, see the section about FabA and FabZ), *cis*-5-dodecenoyl-ACP and *cis*-7-tetradecenoyl-ACP each with malonyl-ACP [72]. Only the last elongation step that leads to the formation of vaccenic acid is catalyzed by FabF [70].

Deletion of the *fabH* gene has been thought to be lethal [73]. However, a recent study has shown that this is only true in a mutant strain that is also SpoT1-negative [74] and only a significant reduction in growth rate and cell size upon *fabH* deletion was reported. Thus, the activity of FabH can only partly be substituted by other enzymes. Regarding the fatty acid profile, deletion of *fabH* leads to enhanced production of C18 species, whereas the amount of C16 species is reduced [74]. Overexpression of *fabH* leads to the opposite effect - at the expense of C18:1, C14 and C16 fatty acids are more abundant than in wild-type cells [75].

Deletion of *fabF* leads to a temperature-sensitive mutant, because the elongation of C14:1 to C16:1 is performed mainly by FabF [66]. Overexpression has been shown to be lethal and a strong increase of malonyl-CoA intermediates has been observed [76]. The lethal effect could partly be resolved by coexpression of *fabD*, and thus, it has been proposed that FabD (which catalyzes the transacylation of malonyl-CoA to malonyl-ACP) forms complexes with FabF or FabH and eventually also with FabB. Overexpression of *fabF* would leave substantially no free FabD proteins and thus the FabD-FabH complex would not be formed. This could hinder the correct FabH activity and block the synthesis of new fatty acids. Malonyl-ACP would easily be converted to malonyl-CoA, which accumulates, as has been shown [76].

Deletion of *fabB* in *E. coli* leads to auxotrophy for unsaturated fatty acids [77]. Overexpression is only suitable to enhance the unsaturated fatty acid proportion if *fabA* is also overexpressed [78]. However, a significant increase in total fatty acid content is not achieved by *fabA* and *fabB* overexpression and also the co-production of ‘TesA increases the total fatty acid content only by 50% [78].

The enzyme FabH catalyzes the first condensation reaction and its activity is inhibited by high levels of long-chain acyl-ACP esters [79], which accumulate when the rate of membrane synthesis slows down. Furthermore, the accumulation of long-chain acyl-ACP esters redirects the activities of FabF and FabB towards the formation of acetyl-ACP on the expense of malonyl-ACP. Transesterification of acetyl-ACP to acetyl-CoA is catalyzed by FabH, when the enzyme is bound to long-chain acyl-ACP [80]. As *fab*F and *fab*H are cotranscribed with the *E. coli fab*-cluster, their transcriptional regulation will be discussed in Section ‘Promoters of the *fab*-operon’. Transcription of *fabB* is modulated by the repressors FadR and FabR (detailed in ‘Transcriptional regulation by FadR and FabR’).

### FabG: 3-ketoacyl-ACP reductase

Following the condensation reaction, the resulting 3-ketoacyl-ACP is reduced to 3-hydroxyacyl-ACP at the concomittant expense of NADPH and H^+^. This reversible reaction is catalyzed by FabG. FabG of *E. coli* was first purified and analyzed by Toomey and Wakil [81], who found that it is active over a wide range of different 3-ketoacyl-ACPs. Activity with acetoacetyl-CoA has also been demonstrated, although the rate of reduction was only 2% of the rate with the corresponding ACP ester. NADH is not used as a coenzyme [81]. Also 3-ketoacyl-CoA with longer chains are suitable substrates [82]; thereby, the FabG protein is also involved in the engineered biosynthesis of polyhydroxyalkanoates consisting of medium chain-length constituents [82-84]. In *E. coli* the FabG activity in fatty acid biosynthesis cannot be substituted by any other enzyme [64,85]. Homologous expression of *fabG* in *E. coli* increases the content of C16:0 and C18:0 acids by two and three times [86]. The transcriptional regulation of *fabG* is discussed in Section ‘Promoters of the *fab*-operon’.

### FabA and FabZ: 3-hydroxyacyl-ACP dehydrase

The enzymes encoded by *fab*Z and *fab*A in *E. coli* perform the dehydration of 3-hydroxyacyl-ACP. Moreover, FabA subsequently isomerizes *trans*-2-decenoyl-ACP into *cis*-3-decenoyl-ACP [87], which is the first reaction towards the synthesis of unsaturated fatty acids. Under physiological conditions, these reactions can be catalyzed in both directions depending on the substrate and product concentrations.

In earlier studies FabA was thought to only act in the synthesis of unsaturated fatty acids, because a defect in *fab*A led to auxotrophy of unsaturated fatty acids [88-90]. However, further investigations showed that *fab*A overexpression increases the amount of saturated fatty acids [91] and that FabA also catalyzes the dehydration of saturated 3-hydroxyacyl-ACPs with different chain lengths [92]. The explanation for the saturated fatty acid accumulation in a FabA-overproducing strain is, that *cis*-3-decenoyl-ACP is not further reduced to decenoyl-ACP at an appropriate rate and thus, reacts in the reversible reaction and re-enters the cycle for the synthesis of saturated fatty acids [91]. Only a strain that co-overexpresses *fabA* and *fabB* will produce an enhanced proportion of unsaturated fatty acids [78]. FabA can also dehydrate 3-hydroxydecanoyl-CoA with an activity of 11% in comparison to 3-hydroxydecanoyl-ACP [93].

The second enzyme of *E. coli* that catalyzes the dehydration of 3-hydroxyacyl-ACP intermediates was detected in 1994 [94] and was designated as FabZ. It was found that the dehydration of 3-hydroxymyristoyl-ACP is mainly performed by FabZ, and thus it was suggested that disruption of *fabZ* leads to an enhanced pool of 3-hydroxymyristoyl-ACP [79,94]. Besides this reaction, FabZ exhibits activity for all 3-hydroxyacyl-ACP with shorter chain-length [79]. Homologous overexpression of *fabZ* results in an about 2-fold increase in palmitic acid and stearic acid levels [86]. In *E. coli*, *fab*Z is part of the lipid A cluster, whereas *fab*A is not transcribed together with other fatty acid or lipid-metabolism genes. However, *fabA* is under control of the regulators FadR and FabR (detailed in ‘Transcriptional regulation by FadR and FabR’).

### FabI: enoyl-ACP reductase

In *E. coli* there is a single enoyl-ACP reductase that performs the last step in each fatty acid biosynthesis cycle, which makes the gene essential if no external fatty acids are fed [95,96]. The enzyme, which is encoded by *fabI*[97], catalyzes the reduction of 2-enoyl-ACP to fatty acyl-ACP at the expense of NADPH + H^+^ or NADH + H^+^.

As the two preceding steps in fatty acid biosynthesis are reversible under physiological conditions, FabI plays a determinant role in completing each elongation cycle [98]. This important role makes the enzyme a suitable candidate for posttranslational regulation. The enzyme is severely inhibited by low concentrations of palmitoyl-CoA (with a *K*_i_ of 3.3 μM in an NADH-dependent enzyme assay and 1.6 μM with NADPH), presumably to prevent the energy-expensive biosynthesis of fatty acids, when they are already available [95]. The enoyl-ACP reductase is also inhibited by its product palmitoyl-ACP, but at about 50 times higher concentrations [79,98]. The *fab*I gene in *E. coli* is not part of any cluster that concerns lipid synthesis. Overexpression of the *fabI* gene does not result in any growth defect [99] but also does not increase the cellular lipid, palmitic acid or stearic acid content [86].

### ACP, ACP synthase and ACP phosphodiesterase

The acyl carrier protein is encoded by *acpP*, which in *E. coli* is part of the *fab*-cluster. To get the physiologically active form, a phosphopantethein group is attached to a serine of the translated *apo*-ACP by the action of the ACP synthase (AcpS). In *E. coli* an ACP phosphodiesterase also exists (AcpH) that cleaves the phosphopantethein residue of [100,101]. The physiological role of ACP is to differentiate fatty acid biosynthesis where all intermediates are bound to ACP from fatty acid catabolism, where all intermediates are CoA esters. In *E. coli*, ACP represents 0.25% of all soluble proteins [102] and thus, belongs to the most abundant proteins. Its absolute requirement has been demonstrated by Goh *et al*. [103], who stopped cell growth by inducible gene-silencing of *acpP*.

Overexpression of *acpS* leads to cessation of cellular growth [104], which is due to strong inhibition of the glycerol-3-phosphate acyltransferase [104,105]. This effect can be slightly resolved by coexpression of *acpH*, because *apo*-ACP is a stronger inhibitor than *holo*-ACP. However, the inhibition of fatty acid biosynthesis has additionally been shown *in vitro* by the supplementation of *holo*- or *apo*-ACP to cell-free extracts of a FFA-producing *E. coli* strain [106]. Coproduction of ‘TesA and *apo*-ACP stops cell growth completely and leads to 5-fold stronger formation of free fatty acids compared to the expression solely of ‘*tesA*[104]. Also the heterologous expression of *acpP* offers some potential, as it has been shown that the expression of *acpP* from *Azospirillum brasilense* alters the *E. coli* fatty acid profile and the content of C18:1 is increased 2-fold at 30°C [107].

A recent study of Battesti and Bouveret [108] has shown evidence for an interaction between ACP and SpoT, a protein that can synthesize or degrade the signal molecule (p) ppGpp (guanosine 5-triphosphate, 3-diphosphate). They suggested a model in which SpoT senses the functionality of the fatty acid biosynthesis and the consumption of acyl-ACP, by the interaction with ACP and mediates the stringent response if fatty acid biosynthesis is impaired. Additionally, they showed that SpoT cannot interact with *apo*-ACP [108].

## Membrane synthesis

In bacteria two systems exist for the formation of 1-acyl-glycerol-3-phosphate; the genes for both are present in *E. coli*[109]. The end product of fatty acid biosynthesis, acyl-ACP, can be activated with an inorganic phosphate group by the action of PlsX, leading to acyl-phosphate, which is subsequently added to glycerol-3-phosphate by the action of PlsY. Alternatively the fatty acid moiety of acyl-ACP can directly be condensed with glycerol-3-phosphate by PlsB. The following steps to synthesize diacylglycerol-3-phosphate and cytosine diphosphate diacylglycerol are performed by PlsC and CdsA. The latter intermediate is then used for the formation of phosphatidylethanolamine, phosphatidylglycerol, cardiolipin or other membrane lipids [110,111].

PlsB of *E. coli* is active with both acyl-CoA and acyl-ACP with similar affinity. The *K*_m_ values for palmitoyl-CoA and palmitoyl-ACP have been determined to be 50 μM and 70 μM [112]. Elevated (p)ppGpp levels, as in the stringent response, seriously reduce the *in vivo* activity of PlsB, whereas the *in vitro* activity is not altered. Accordingly posttranslational inhibition of PlsB by (p)ppGpp has been suggested [113]. As a result, the intracellular level of acyl-ACP increases, which inhibits the fatty acid biosynthesis at several points [79] (see Section ‘Regulation by the stringent response’). Contrary to the data from Heath and coworkers [113], the inhibition of PlsB by (p)ppGpp has been shown *in vitro* by Ray and Cronan [112] with the substrate palmitoyl-CoA. The reaction with palmitoyl-ACP was not affected under the conditions of the enzyme test. Overexpression of the *plsB* gene resolves the inhibition of PlsB by (p)ppGpp and thus, the fatty acid biosynthesis remains active, which leads to the formation of longer cells because the cell division is still affected [113].

Transcription of *plsB* has been found to be antagonistic to transcription of the gene for diacylglycerol kinase (*dgkA*) [114], so that the cell can react to different stresses with either the biosynthesis of new membrane lipids or the modification of the headgroups via degradation to diacylglycerol, phosphorylation and addition of an alternative headgroup. Transcription of *plsB* is inhibited in response to high levels of BasR [114], a regulator protein that mediates iron or zinc stress [115]. If *E. coli* is in an environment that leads to degradation of the lipopolysaccharides or otherwise stresses the cell envelope, the σ^E^ regulon is activated, which leads to induction of *plsB* transcription [114]. During the multiple changes in the transcriptome that are mediated by the stringent response (due to amino-acid starvation) the *plsB* transcription is downregulated [116].

Despite the existence of both systems, PlsX and PlsY or PlsB cannot substitute for a complete loss of the other system in *E. coli*[117]. However, no significant increase in glycerol-3-phosphate acyltransferase activity has been found upon overexpression of *plsX* and *plsY*[118]. Transcription of *plsX* and *plsB* are both inhibited during the stringent response [116,119]. Thus, it was suggested that PlsX and PlsY determine the concentration of acyl-phosphate, which might have a further regulatory function [117].

## Fatty acid degradation

To metabolize fatty acids, they must be activated to acyl-CoA esters. If fatty acids are the exogenous carbon source, they bind to the transporter protein FadL. By a conformational change, a pore is opened and the diffusion of fatty acids into the periplasm is enabled [120]. Disruption of *fadL* impaires growth on oleate [121]. Transport from the periplasm to the cytosol is performed by FadD and is coupled to the acyl-CoA ester formation at the expense of ATP [122]. As has been shown recently, FadD also uses free fatty acids that are cleaved from membrane lipids and the formed acyl-CoA is consumed via the *β*-oxidation pathway. Consequently, a *fadD-*disruption mutant accumulates free fatty acids in the cytosol [123] and apart from this, is unable to grow on oleate as a sole carbon source [121]. Homologous overexpression of *fadD* enables *E. coli* strains to grow on fatty acids with medium chain-length and enhances the transcription of the *fadE* and *fadBA* genes [124]. The affinity of FadD for medium chain-length fatty acids can be enhanced by directed mutagenesis [125].

The degradation of acyl-CoA compounds proceeds in a cycle that reverses the steps of the fatty acid biosynthesis, resulting in the release of one unit of acetyl-CoA in each cycle. FadE catalyzes the oxidation of acyl-CoA to enoyl-CoA with a concomitant reduction of FAD to FADH_2_. Disruption of *fadE* disables the ability of *E. coli* to grow on dodecanoate or oleic acid as the sole carbon source [121]. The enzyme FadB performs the hydration of enoyl-CoA to 3-hydroxyacyl-CoA and further oxidizes this intermediate to 3-ketoacyl-CoA [126]. The *β*-ketothiolase FadA catalyzes the last step in the cycle in which acetyl-CoA and an acyl-CoA (reduced by two carbon atoms) are formed [127]. The final cleavage of acetoacetyl-CoA is performed by YquF [127,128]. The catabolism of unsaturated fatty acids additionally involves the proteins FadH (2,4-dienoyl-CoA reductase) [129] and probably also FadM (thioesterase III) [130]. However, the exact role of FadM is still not clear, as its transcription is quite strong during growth on glucose. This suggests that the protein function is not limited to fatty acid degradation [131].

In anaerobic growth on fatty acids *E. coli* possesses some alternative proteins, namely YfcYX (homologs of FadBA) and YdiD (FadD homolog). An alternative FadE protein (YdiO) is also suggested. These two sets of proteins are not completely selective for aerobic or anaerobic conditions, for example, YfcYX can partially compensate for a loss of FadB and FadA [132-134]. In the anaerobic fatty acid degradation pathway, nitrate is used as the final electron acceptor. Repression of the transcription of the genes for the aerobic cycle is mediated by the ArcA/ArcB two-component system [135]. For more detailed information about the bacterial *β*-oxidation, the reader is referred to recent review articles [136,137].

All genes of the aerobic fatty acid degradation cycle are under transcriptional control of the repressor protein FadR (for details see Section ‘Transcriptional regulation by FadR and FabR’), which releases the DNA upon binding of long-chain acyl-CoA, and thus enables transcription of the *β*-oxidation genes [138,139]. Furthermore, the fatty acid degradation cycle is under positive control by the cyclic adenosine-monophosphate (cAMP) receptor protein-cAMP complex (CRP-cAMP), so that at least *fadL*, *fadD* and *fadH* are upregulated when glucose is limited [140]. In addition, the upregulation of several genes of the *β*-oxidation involves the sigma factor σ^S^ (RpoS) [141], as detailed in Section ‘Regulation by the stringent response’.

As discussed in the Section about free fatty acid production, *fadL*, *fadD* or *fadE* have been deleted to prevent product uptake or utilization in fatty acid or related biofuel-producing strains [26,142,143]. All of them have been shown to increase the yields of the desired product, at least in some of the studies.

## Regulation in *E. coli*

**Figure 3:**
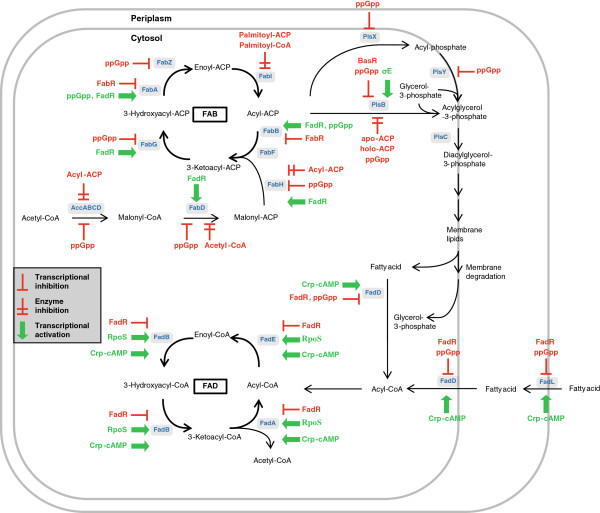
**Regulation of the lipid metabolism.** Activating compounds are colored green, and repressing compounds are shown in red. FAB, fatty acid biosynthesis; FAD, fatty acid degradation.

In *E. coli* several genes for fatty acid biosynthesis or degradation are controlled at the transcriptional level. The main transcription factors are the proteins FabR and FadR, but also other regulators, notably the signal molecule (p)ppGpp are involved (Figure 3).

### Transcriptional regulation by FadR and FabR

The regulator FadR represses the transcription of all genes that code for proteins of the *β*-oxidation cycle [136,140] and via activation of the repressor protein IclR, also of the glyoxylate shunt [144,145]. FadR repression is due to binding to the promotor sites, which is released by interaction with long-chain acyl-CoA esters that accumulate only if external fatty acids are taken up or if phospholipids from the membrane are degraded [138,139,146]. Since the uptake of fatty acids is mediated by FadL and FadD, the corresponding genes are only partially repressed by FadR [140] and additionally activated by CRP-cAMP, under glucose limiting conditions. In the presence of glucose, transcription of the *fad*-genes occurs only at a low level, even when fatty acids are also available [147]. Besides the negative control of the *fad*-regulon, FadR also acts as a positive regulator for the transcription of *fabA*, *fabB* and the operon *fabHDG*[138,139,148,149], and thus, is the activator for the formation of both unsaturated and saturated fatty acids.

In a mutant strain of *E. coli* that synthesizes no functional FadR, all *fad*-genes are derepressed in addition to the activation under glucose limiting conditions. The resulting phenotype is such that the strain can grow on fatty acids with medium chain-length, whereas the wild-type can use them only if long-chain fatty acids are also present and sequester FadR [150]. Due to the lacking induction of the transcription of *fabA* and *fabB*, a *fadR* mutant strain has a roughly 30% lower concentration of unsaturated fatty acids [140,151]. On the contrary, the homologous expression of *fadR* in a fatty acid-producing strain of *E. coli* can increase the yield of saturated and unsaturated fatty acids significantly [152].

In the study of Farewell *et al*. [153] a FadR mutant was investigated that cannot be derepressed by long-chain fatty acids. Probably owing to the impairment of unsaturated fatty acid synthesis, this mutant exhibited a low survival rate during long-time cultivations.

Besides their regulation by FadR, *fabA* and *fabB* are further under control of the fatty acid biosynthesis regulator FabR [154,155]. More recently it has been shown that FabR senses the composition of the cytosolic fatty acid pool. In complex with unsaturated fatty acyl-ACP the binding and thus, the repression of *fabA* and *fabB* is strengthened, whereas the binding is weakened when FabR is bound to saturated fatty acyl-ACP [156]. According to the study of Feng and Cronan [157], FabR is the main regulator for *fabB*, whereas the transcription of *fabA* is more strongly influenced by the action of FadR.

### Promoters of the *fab*-operon

Many of the genes coding for enzymes of fatty acid biosynthesis in *E. coli* are organized in a cluster and under control of different promoters. This so called *fab*-cluster comprises the genes *plsX*, *fabH*, *fabD*, *fabG*, *acpP* and *fabF. PlsX* is the first gene of this cluster; however, its own promoter is rather weak so that about 60% of all transcripts containing *plsX*-mRNA result from promoters located further upstream [64,158]. This longer mRNA contains transcripts of *yceD* (coding for an uncharacterized protein) and *rpmF* (coding for the 50S ribosomal subunit protein L32) so that coordinated regulation of these proteins and fatty acid biosynthesis seems possible [159].

The genes *fabH*, *fabD* and *fabG* encode for proteins that catalyze subsequent steps in the initiation of fatty acid biosynthesis. They are transcribed by a strong promoter within the upstream region of *plsX* and a weak promoter upstream of *fabD*[64,160]. The strong promoter has been investigated unravelling a 4-fold downregulation of transcription upon amino-acid starvation [160], which is known to induce the synthesis of (p)ppGpp. Furthermore, the transcription of *fabHDG* at a normal level requires induction by FadR [149]. The weaker promoter is thought to complement for polar effects [64]. Lacking a promoter directly upstream of the coding sequence, *fabG* mRNA seems to be formed by processing of the longer transcripts [64]. The last two genes of the *fab*-*cluster*, *acpP* and *fabF*, each possess a strong promoter and for the former, no control by the growth rate or by FadR could be found [64].

### Regulation by the stringent response

Many studies have shown that fatty acid biosynthesis in *E. coli* correlates with the growth rate [74,161,162], but the underlying mechanism was only partially investigated. Of great importance is the concentration of the global regulator (p)ppGpp, which in *E. coli* can be synthesized by the action of RelA and either hydrolyzed or synthesized by SpoT. Elevated concentrations of (p)ppGpp, in combination with the regulator protein DksA [163,164], influence the stability of the RNA polymerase complex. As a result, the respective genes are activated or inactivated. As enhanced (p)ppGpp concentrations tend to destabilize σ^70^ promoters, the use of sigma factors other than σ^70^ is facilitated, thereby further extending the alteration of gene expression [116,119,165,166]. The resulting changes of the cellular processes are referred to as the stringent response [167].

One sigma factor that is upregulated by elevated concentrations of (p)ppGpp in the stationary phase is RpoS [168]. RpoS is involved in multiple stress responses, including UV, acid, heat, oxidative stress or starvation [169-172]. With respect to the fatty acid metabolism, the induction of *fadA*, *fadB*, *fadE* and *fadH* expression by RpoS is of interest [141]. For additional information about the regulation and influences of RpoS, the reader is referred to a recent review article [173].

In cells growing under optimal conditions the concentration of (p)ppGpp is very low; however, it can be increased by amino-acid starvation [174], carbon-source depletion [175], phosphate limitation [176,177], iron limitation [178] or inhibition of fatty acid biosynthesis [108,179]. In *E. coli*, RelA is associated with the ribosomes and senses the binding of uncharged tRNAs (during amino-acid limitation), upon which the synthase activity of RelA is induced [174,180,181]. Furthermore, the degradation of (p)ppGpp by SpoT is reduced 5-fold, leading to a more than 20-fold increase in (p)ppGpp concentration. Glucose starvation triggers an approximately 5-fold increase of the (p)ppGpp concentration [176,182], which is achieved by a strong inhibition of the hydrolase activity and a decrease of the synthesis of (p)ppGpp to 30%. As SpoT permanently interacts with the acyl carrier protein [108], it is likely that the degradation or synthase activities of SpoT are influenced by the difference in the charge of ACP, similar to RelA. The hereby provided link between fatty acid biosynthesis and (p)ppGpp concentration might be responsible for the increase in (p)ppGpp concentration under carbon limitation.

Elevated (p)ppGpp concentrations have direct and indirect effects on the biosynthesis of fatty acids. Fastest is the inhibition of enzymes, as shown *in vivo* for PlsB [113] and *in vitro* for FabZ [183]. On the level of transcription, a 4-fold decrease of the promoter of *fabH*, which controls *fabH*, *fabD* and *fabG*, is triggered by amino-acid starvation [160]. Additionally, the transcription of *plsB*, *accBC* and *fadR* is downregulated [116,119,149], whereas *fabA*, *fabB*, *cfa* and *ybhO* appear to be upregulated. The respective gene products are involved in the synthesis of stearic acid, unsaturated and cyclopropane fatty acids and cardiolipin [119]. These processes are typically for *E. coli* cells entering the stationary phase [153,184,185]. However, the enhanced expression of *cfa* is due to its σ^S^-dependent promoter [168]. The same or a similar mechanism might also explain the upregulation of *fabA* and *fabB*, which stands in contrast to the decreased synthesis of their activator, FadR, during the stationary growth phase.

The inhibition of PlsB leads to an increase in the amount of long-chain fatty acyl-ACP, which in turn inhibit FabH, FabI [79] and the acetyl-CoA carboxylase [59]. An additional result of acyl-ACP accumulation is the modified activity of FabH, FabF and FabB, which leads to the degradation of malonyl-ACP to acetyl-CoA via the intermediate, acetyl-ACP. Overexpression of *plsB* partially relieves the inhibition of the fatty acid synthesis, leading to very long cells in the stationary phase [113].

As for the genes coding for enzymes of the *β*-oxidation pathway, the expression of *yfcX* and *fadE* is enhanced during the stringent response. On the contrary, the expression of *fadD* during stringent response of the wild-type was lower than in the mutant control strain that was not able to accumulate (p)ppGpp upon amino-acid starvation [166]. In this context one should keep in mind that the expression of the enzymes of the fatty acid degradation cycle is regulated by FadR [138,139], whereas the expression of *fadD* can be induced by CRP-cAMP [140] (see also Section ‘Fatty acid degradation’).

Further impacts of the high (p)ppGpp concentration during the stringent response are the inhibition of the genes for tRNAs, rRNAs and ribosomal proteins [186,187], as well as of the initiation factor IF2 [188]. A recent study by Edwards *et al*. [189] also found interactions between the carbon-storage regulators CsrA, CsrB and CsrC with the regulators of the stringent response.

### Regulation according to the growth conditions

Long-chain fatty acids can be metabolized by *E. coli*; however, carbohydrates are the preferred carbon sources [140]. Aerobic growth on glucose is accompanied by a missing transcriptional induction of the *β*-oxidation genes, by CRP-cAMP [140,147], and by the repression by FadR [136,140]. Since the repression of *fadD* and *fadL* is less stringent, fatty acids can be taken up in small amounts [140] and relieve the binding of FadR to the respective promoters [138,139]. Due to the similar affinity of PlsB towards fatty acyl-CoA, as towards fatty acyl-ACP, the former can directly be used for the membrane biosynthesis [112]. If glucose (and any other suitable carbohydrate carbon source) is also missing, the concentration of cAMP increases and the complex CRP-cAMP is formed, which binds to the promoters of the *β*-oxidation genes and induces their transcription [140].

Under anaerobic conditions, the situation is somewhat different: Transcription of *fadA*, *fadB*, *fadE*, *fadD* and, to a lesser extend, *fadL* is inhibited by the regulator ArcA [135], and the proteins FadK, YfcY, YfcX, YdiO and YdiD are responsible for the anaerobic fatty acid degradation, if a more suitable carbon source is not available [132]. Under these conditions, uptake of long-chain fatty acids by FadL is very slow and activation by FadD is not possible. However, FadK, which replaces FadD under anaerobic conditions, has a low activity towards long-chain fatty acids. Consequently, anaerobic growth on oleic acid is possible but is very slow [133]. In contrast to long-chain fatty acids, the transport of fatty acids with a medium or short chain-length does not require the activity of FadL [132,151]. Also the activity of the other proteins of the anaerobic *β*-oxidation, for short and medium chain-length fatty acids, is sufficient to enable a robust growth on these substrates under anaerobic conditions [132].

## Production of free fatty acids

The use of *E. coli* and other microorganisms for the production of free fatty acids was initiated by the discovery that the periplasmic enzyme thioesterase I (TesA) deregulates the tight product inhibition of fatty acid synthesis, when expressed as a cytosolic enzyme (‘TesA) [190]. This enzyme cleaves the fatty acyl-ACP, and with a considerably lower activity the fatty acyl-CoA thioester bond also. The resulting free fatty acids accumulate in the late exponential and in the stationary phase and are mostly released to the culture medium [190].

Since fatty acids are very energy-dense, produced in relatively large amounts and in every organism, they represent a suitable target for the development of single-cell oils. Also the use of alternative carbon sources has been demonstrated in a variety of microorganisms. With increasing attention towards the search of sustainable energy sources, many studies have been performed in the last 15 years with the aim of utilizing fatty acid biosynthesis for biofuel production. However, due to the strict regulation of this pathway much basic research is still needed to improve the yields of free fatty acids or related products. Besides of *E. coli*, fatty acid overproduction has been established in cyanobacteria [23,191] and yeast [192].

### Thioesterase expression and physiological consequences

As shown in Table 1, every strategy that yielded a concentration of more than 0.2 g l^-1^ fatty acids used a cytosolic thioesterase from *E. coli* or from a different organism. By the use of different thioesterases, the product can be considerably altered with respect to yield, fatty acid chain-length and degree of unsaturation [143,193-195]. However, the expression level of any thioesterase must be tuned carefully, because already low levels increase the fatty acid titer significantly, and too strong a thioesterase activity has been shown to impair FFA production, both in *in vitro* and *in vivo* experiments [106,196]. In addition, a high titer of FFA in the culture medium can also cause severe defects in the cellular viability. Desbois and Smith [197] summarized the antibacterial actions of FFA, ranging from membrane lysis and interruption of the electron transport chain to possible interferences with membrane proteins or nutrient uptake. Concerning the physiological effects of endogenous FFA overproduction, it has been shown that thioesterase overexpression can alter the degree of saturation of the membrane lipids in *E. coli*[193] and in *Synechococcus elongatus*[191]. Additional effects are the induction of stress responses and reduced membrane integrity and viability of the production strains [193,198]. In the study of Lennen and coworkers [199], an improved fatty acid export system has been suggested to improve viability, and several components of the *E. coli* system have been identified and investigated. Deletion of the gene of the fatty acid transporter FadL has already been tested in combination with *tesA* overexpression [142] and gave promising results.

**Table 1:** Efficiency of genetic modifications

**Variable**	**Background**	**Improvement of the total yield (x-fold)**	**References**
Thioesterase - overexpression	Wild-type	12-fold to 35-fold (1)	[26,195,202]
Δ*fadD*	Wild-type	3-fold to 10-fold (1)	[143,194,202]
Δ*fadE*	Wild-type	5-fold (1)	[196]
Thioesterase - overexpression	Δ*fadD*	1.5-fold to 11.5-fold (2)	[26,143,202]
Thioesterase - overexpression	Δ*fadE*	4-fold (2)	[196]
Δ*fadD*	Thioesterase overexpression	2-fold (2)	[26]
Δ*fadE*	Thioesterase overexpression	3-fold (2)	[26]
*accABCD*	Δ*fadD* or Δ*fadD* + Thioesterase overexpression	1.1-fold to 1.33-fold (2)	[143,202]
*fabF*	Thioesterase overexpression + Δ*fadE*	15 fold diminished or 3-fold enhanced (2)	[152,196]
*fabZ*	Thioesterase overexpression + Δ*fadD* or Δ*fadE*	3-fold enhanced or no change (2)	[196,204]
*fabG*; *fabZ*; *fabI*	Thioesterase overexpression + Δ*fadE*	1.5-fold (2)	[196]
*fabA*	Thioesterase overexpression + Δ*fadE*	1.1-fold (2)	[152]
*fabB*	Thioesterase overexpression + Δ*fadE*	2.3-fold (2)	[152]
*fabBA*	Thioesterase overexpression + Δ*fadE*	1.7-fold (2)	[152]
*fadR*	Thioesterase overexpression + Δ*fadE*	7.4-fold (2)	[152]

(1) Wild-type = 0.02 g l^-1^[143]. (2) Compared with the reference strain of the same study. The table is sorted according to the overexpression or deletion of a single gene (variable). For calculation of the yield improvement, we compared the final fatty acid concentration of the background strain with the same strain plus deletion or overexpression of the respective gene. Thioesterases from different organisms have been tested, but were always expressed as a cytosolic enzyme. All other genes in this table were derived from *E. coli*.

Of importance for the microbial production of biofuels are strategies to enhance the tolerance of *E. coli* towards organic solvents, as performed by Oh and coworkers [200]. Deletion of *fadR* resulted in an enhanced proportion of saturated fatty acids in the membrane of *E. coli*, as has been observed in previous studies [140,151]. The higher grade of saturation made the membrane less permeable for organic solvents. By deletion of *marR*, the repressor of *marA* expression, the multidrug resistance of *E. coli* was permanently induced. Besides others this led to the constitutive expression of *tolC*, *acrA* and *acrB*. The gene products build an efflux system for organic solvents and thus enhance the survival of *E. coli* in presence of high concentrations of organic solvents. Interestingly, these genes are exactly the same genes that had been proposed by Lennen and coworkers [199] for improved fatty acid export. A combination of both deletions (*fadR* and *marR*) led to an even higher tolerance of organic solvents, compared to the single deletions [200]. However, *fadR* deletion may not be ideal, if one aims at the production of fatty acids. Hence, a combination of *marR* deletion and the improved synthesis of saturated fatty acids, for example, by overexpression of *fabA*[91] or *fabZ*[86], appears to be promising.

### Deletion of *β*-oxidation genes

To prevent product degradation, many studies have been performed in a strain that was inhibited in fatty acid *β*-oxidation. The main target for deletion was *fadD*[26,106,143,190,194,201-203], whereas deletion of *fadE* was mainly done when the activation of the FFA to fatty acyl-CoA esters was necessary for further product processing [26,142,152]. Although most studies found FFA levels enhanced upon (partial) deletion of the *β*-oxidation pathway or did not control the success of this deletion, Cho and Cronan [190], as well as Liu and coworkers [142] did not detect a positive effect when thioesterase overexpression was combined with the deletions of *fadD*, *fadE* or *fadL* (to impair re-uptake of FFA). In these studies it was suggested that the *β*-oxidation pathway has not the capacity to cope with the strong FFA production. An alterative explanation might be that the positive control of *fadL*, *fadD* and *fadH* by the cAMP receptor protein-cAMP complex [140] was limiting in some of the performed studies, which might be caused by different cultivation conditions. In contrast, it seems unlikely that the negative control via the FadR repressor (released upon acyl-CoA binding) differed in the studies where the *fadD* or *fadL* genes were not deleted.

### Investigation and remodeling of the whole pathway

In order to improve FFA production on a broad scale, a computational model of the *E. coli* metabolism has been used, and several deletions in the glycolysis or tricarboxylic acid cycle have been investigated along with the overexpression of genes of fatty acid biosynthesis [204]. Deletion of the genes responsible for acetate formation has been tested to improve malonyl-CoA titers [61] or FFA productivity [194,201,205]. This strategy clearly reduced acetate formation; however, in the two latter studies the reduction of acetate formation did not enhance FFA yields. Instead, Zhang and coworkers [194] state that the acetate formation is already diminished in efficient FFA producers. This is also interesting with respect to the pH of the medium, as *E. coli* production strains tend to slightly increase the pH, instead of decreasing it as wild-type cells [194].

An alternative way to investigate the production of FFA as a whole is the reconstruction of the pathway under controlled conditions *in vitro*. In the study of Liu *et a*l. [106] cell extracts of *E. coli* production strains were used to determine the concentrations of NADPH and malonyl-CoA, which enabled half-maximal reaction velocity. For NADPH a *K*_m_ value of 34.7 μM was calculated, and based on an estimated concentration of NADPH in the cytosol of 100 to 200 μM [206-208] it was concluded that this cofactor should not be limiting. In contrast, the calculated *K*_m_ value for malonyl-CoA of 15.7 μM exceeds the estimates of Davis *et al*. [60] and Bennet *et al*. [206], who give a cellular concentration of malonyl-CoA of less than 5 μM. Consequently, the addition of malonyl-CoA or acetyl-CoA-carboxylase to cell free extracts enhanced the *in vitro* reaction velocity of fatty acid biosynthesis [106]. In addition, the overexpression of the acetyl-CoA carboxylase was also investigated several times in combination with other genetic modifications, and successfully enhanced FFA production [60,106,143]. However, Acc overexpression alone was not sufficient for FFA production in a Δ*fadD* background [202]. A detailed study of how to improve the malonyl-CoA concentration in the cytosol has been performed by Zha and coworkers [61]. By deletion of acetate formation pathways, heterologous expression of Acc from *Corynebacterium glutamicum* and the *E. coli* acetyl-CoA synthase, 16-fold higher malonyl-CoA content was achieved.

For those who wish to improve the substrate or cofactor supply, Yu *et al*. [196] have tested higher concentrations of acetyl-CoA (0.5 mM), malonyl-CoA (1.5 mM), NADH (1 mM), NADPH (1.5 mM), NAD^+^ (5 mM) and NADP^+^ (5 mM) in an *in vitro* reconstitution experiment of fatty acid biosynthesis. With these concentrations, no inhibition was observed. In contrast, Liu and coworkers [106] found a strong inhibitory effect of high concentrations of *apo*- and *holo*-ACP on *in vitro* fatty acid synthesis. The limit for a beneficial effect has been determined to be 32 μM for both *apo*- or *holo*-ACP [196]. This finding is in agreement with the study of Keating *et al*. [104], who found that overexpression of *tesA* and *acpP* strongly inhibits the growth of *E. coli*. Coexpression of *acpS* relieved this phenotype only slightly, suggesting that both *apo*- and *holo*-ACP are also inhibitory *in vivo*.

With respect to the other enzymes of fatty acid biosynthesis, it was found that FabA, FabB, FabD, FabF, FabG, FabH, FabI and FabZ occur in about equal concentrations in wild-type *E. coli* cells, whereas TesA and *holo*-ACP proteins are considerably more abundant [196]. To get an even better understanding of potential candidates for overexpression, an *in vitro* assay was performed using purified enzymes, with fixed concentrations. When all enzymes were used in a concentration of 1 μM and 10 μM for ACP and TesA respectively, a further enhancement of the concentrations of FabA, FabB, FabD or FabG did not result in an increase of activity. FabF and FabH (the latter less pronounced) inhibited the enzymes of FAB at higher concentrations, whereas FabI and FabZ enhanced the FAB activity 2- and 6-fold, respectively, when added at a concentration of 10 μM [196]. Some of these data were confirmed *in vivo* (see Table 1), for example, the lack of enhanced FFA production upon (co-)overexpression of FabA [78,152], whereas coexpression of FabB doubled the FFA production in an *E. coli* strain with a *fadD*-deletion and overexpression of *tesA*[152]. The role of FabF remains even more controversial. Due to the strong inhibition of the *in vitro* assay, Yu *et al*. [196] tested coexpression of the *fabF* gene in their production strain and could also detect a strong decrease in FFA titer. On the contrary, Zhang *et al*. [152] enhanced the FFA yield of their production strain nearly 3-fold upon *fabF* overexpression. However, they observed a higher FFA yield, when *fabF* was expressed at a lower rate. Both studies were performed using a *fadE* deletion mutant of *E. coli* and strong *tesA* and *fabF* overexpression. Apart from the use of different plasmid systems, Yu and coworkers [196] coexpressed an additional thioesterase from *Cinnamomum camphorum* in both *in vitro* and *in vivo* experiments. This thioesterase has activity towards fatty acids of a chain length ranging from 12 to 18 carbon atoms [209]. It is also noteworthy, that overexpression of *fabF* has a lethal effect in a strain that does not overproduce FFA [76] (compare to the Section about FabB, FabF and FabH).

Similar discrepancies can be found in the literature on *fabZ* overexpression. Upon co-overexpression of *fabZ*, the FFA titer was enhanced nearly 3-fold in a *fadD* deletion mutant with expression of a thioesterase from *Ricinus communis*[76]. In contrast, in the study of Yu and coworkers [196], enhanced levels of FabZ improved the rate of fatty acid biosynthesis only *in vitro*, whereas *in vivo* the combined co-overexpression of *fabZ*, *fabI* and *fabG* was necessary to outperform the control strain with *fadE* deletion and overexpression of *tesA* and the thioesterase from *C. camphorum*. Taken together these results indicate that overexpression of more than one enzyme of the FAB are much more likely to improve FFA production of already existing production strains.

### Altering the regulation or pathway direction

A promising addition to the overexpression of genes that are involved in fatty acid biosynthesis offers the use of regulatory mutants. A possible target to improve the FFA yields is the carbon-storage regulator, which consists of the CsrA protein and the non-coding RNAs CsrB and CsrC [210]. CsrA acts as a posttranscriptional inhibitor or activator by binding to the 5’-untranslated sequence of target mRNAs [211]. This binding can be prevented by the interaction with CsrB or CsrC that consist of several CsrA binding sites and sequester this protein [212]. In the study of Edwards *et al*. [189] 721 transcripts have been identified that copurify with CsrA, which regulates cellular processes such as glycolysis, glycogen formation or the stringent response. McKee *et al*. [213] have used *csrB* overexpression to enhance the productivity of a *tesA* expression strain. Besides the nearly doubled FFA production, a concomitant reduction in acetate formation was observed.

A more obvious candidate to alter the regulatory network is the repressor of fatty acid degradation, FadR. In an *E. coli* strain with a *fadE* deletion and with *tesA* overexpression, coexpression of *fadR* resulted in a more than seven-fold enhanced FFA production [152]. Due to the induction of *fabA* and *fabB*, the coexpression of *fadR* leads to an increase of the unsaturated fatty acid (UFA) content from 13% to 43% in the production strain.

Deletion or disruption of the *fadR* gene leads to a constant expression of the *β*-oxidation genes. This enables *E. coli* to grow aerobically on fatty acids with medium chain-length [214]. The expression can further be enhanced by a mutation in the cAMP receptor protein (crp*) that leads to a deregulated catabolite repression [215]. By the deletion of *arcA*[135] and a mutation in the regulatory gene *atoC*, aerobic growth of *E. coli* on fatty acids with short chain-length is possible [216,217]. The aforementioned mutations in *fadR*, *crp*, *arcA* and *atoC* have been used in combination with the deletion of fermentative pathways and overexpression of the genes *fadB*, *fadA* and *fadM* for a functional reversal of the fatty acid degradation cycle with the aim to produce FFA from non-related carbon sources [201]. As this way does not need the energy-consuming conversion of acetyl-CoA to malonyl-CoA, the theoretical yield for FFA production from glucose can be increased from about 36% (g g^-1^) to 43% (g g^-1^) [218].

### Process optimization

Although many studies have focused on the engineering of an efficient production strain, they were mostly performed in a batch mode. If investigated, fed-batch fermentations have significantly increased the productivity, and titers of 2.5 g l^-1^ to 7 g l^-1^ have been achieved [106,143,201]. Due to the growth inhibitory effect of high concentrations of fatty acids in the culture medium and to the fact that the product must be somehow purified, Liu and coworkers [142] have applied an extraction unit to their fed-batch fermentation system. Beginning 10 hours after induction, the culture volume was pumped through a tributylphosphate phase at a rate of 0.8% per minute (volume for extraction per volume cultivation medium). After passage of the tributylphosphate phase, the culture medium was pumped back into the fermenter vessel. By this process, a total fatty acid production of roughly 9 g l^-1^ was achieved [142].

Although fed-batch fermentations have advantages over batch cultures, continuous fermentations offer an even higher potential, because the cells can be kept under optimal conditions and in the most suitable growth phase. With the aim of FFA production, continuous cultivations of an *E. coli* strain with replacements of *fadD*, *fadE* and *fadAB*, each by one copy of the thioesterse gene from *Umbellularia californica*, have been performed [203,204]. Limitation of carbon, nitrogen or phosphate source has been applied with phosphate limitation enabling the best results. With respect to the carbon source (glucose) a conversion rate to FFA of 0.1 (g g^-1^) has been achieved, and the highest biomass-specific productivity was 0.068 g FFA per g cell dry weight per hour [203].

## Production of FAAE

The production of FAAE has been the focus of several studies in recent years. It relies on the microbial production of fatty acids and of a short chain-length alcohol. After the fatty acids are activated to acyl-CoA the formation of an ester bond with an alcohol is performed enzymatically. For this reaction, nearly all studies performed so far used the promiscuous wax ester synthase/acyl-CoA:diacylglycerol acetyl transferase (WS/DGAT; AtfA) of *Acinetobacter baylyi* ADP1. This enzyme has been shown to exhibit activity with an extraordinary wide range of alcohols and fatty acids [219,220]. As alcohol moiety ethanol is preferred due to its low toxicity for the production organism, compared to methanol or butanol and the ease of its microbial production. The activity of the AtfA towards ethanol is considerably lower than towards the natural substrates diacylglycerol or long chain-length alcohols [219]. An attempt to overcome this possible bottleneck has been the comparison of five different wax ester synthases towards FAEE production in *S. cerevisiae*[221].

The first study to produce FAEE-based (fatty acid ethyl ester) microdieselin *E. coli* has been performed by Kalscheuer *et al*. [24], yielding 1.3 g l^-1^ FAEE by fed-batch fermentation (Table 2). They expressed the *Zymomonas mobilis* pyruvate decarboxylase (*pdc*) and alcohol dehydrogenase B (*adhB*) to produce ethanol. By coexpression of *atfA*, the cells were enabled to form an ester consisting of ethanol and a fatty acid. This study provided the blue print for most subsequent studies to produce FAEE. Optimization of the fed-batch process by Elbahloul and Steinbüchel [222] yielded a maximum FAEE concentration of 11 g l^-1^. However, these two studies used oleic acid that was externally added to the culture medium and did not rely on endogenously produced fatty acids. To produce FAEE from non-related carbon sources, at least a thioesterase (for cleavage of acyl-ACP) and a fatty acid CoA ligase have to be combined with ethanol production and *atfA* expression [26,223]. These studies have also shown a significant increase in FAEE production upon deletion of the *fadE* gene. Fed-batch fermentations to produce FAEE from glucose have been optimized with respect to the medium, time of induction, temperature and feeding rate, by Duan and co-workers [223], yielding a maximal FAEE content of 0.9 g l^-1^.

**Table 2:** **FAEE-producing strains of *E. coli***

**Gene deletion**	**Wax exter synthase and alcohol production**	**Overexpression of other genes**	**Yield (g l**^**-1**^**)**	**Process time (h)**	**Productivity (g l**^**-1 **^**h**^**-1**^**)**	**Process**	**Reference**
-	*atfA*; *pdc*; *adhB*		0.43	48	0.009	batch + oleate	[24]
-	*atfA*; *pdc*; *adhB*		1.28	72	0.018	fed-batch + oleate	[24]
-	*atfA*; *pdc*; *adhB*		11	47	0.234	fed-batch + oleate	[222]
-	*atfA*; ethanol added	*tesA*; *fadD*	0.1	48	0.002	batch	[26]
*fadE*	*atfA*; ethanol added	*tesA*; *fadD*	0.4	48	0.008	batch	[26]
*fadE*	*atfA*; *pdc*; *adhB*	*tesA*	0.037	48	0.001	batch	[26]
*fadE*	*atfA*; *pdc*; *adhB*	*tesA*; *fadD*	0.233	48	0.005	batch	[26]
*fadE*	*atfA*; *pdc*; *adhB*	*tesA*; *fadD*; atfA	0.427	48	0.009	batch	[26]
*fadE*	*atfA*; *pdc*; *adhB*	*tesA*; *fadD*; atfA	0.674	48	0.014	batch with dodecane overlay	[26]
*fadE*	*atfA*; *pdc*; *adhB*	*tesA*; *fadD*; *accABCD*	0.922	72	0.013	fed-batch	[223]
*fadE*	*atfA*; *pdc*; *adhB* (1)	*tesA*; *fadD*; *fadR* (1)	1.5	72	0.021	batch	[191]

(1) Dodecane overlay to prevent fatty acid ethyl ester (FAEE) evaporation. Gene source: *Acinetobacter baylyi* ADP1 (*atfA*); *Zymomonas mobilis* (*pdc*; *adhB*); *Escherichia coli* (all other genes).

Despite the advances in FAEE production derived from non-related carbon sources, the yields are still far too low for commercial purposes. One problem is the instability of production strains that probably results from ethanol accumulation [26,224]. Cultivations of the production strain have been performed at 25 or 30°C [26,223], and the latter study reports that at 37°C no FAEE production was achieved. A more recent study approached the problem of ethanol toxicity by finetuning the expression of all overexpressed genes [152]. This is achieved by coexpression of *fadR* and introduction of FadR binding sites in the strong promoters of all overexpressed genes, despite *tesA*. When FFAs accumulate, the repression of *fadD*, *pdc*, *adhB* and *atfA* by FadR is relieved depending on the amount of FFA. As a result, the formation of ethanol, as well as of acetate could be clearly reduced.

With respect to the use of FAEE as an alternative and renewable energy source, carbon sources must be used that are even cheaper than glucose and do not compete with food and feed production. Attempts have already been made that prove the possibility of using hemicelluloses or pretreated switchgrass for FAEE production by fermentation of *E. coli*[26,225].

Besides FAEE production, the biosynthesis of fatty acid methyl esters has also been performed in *E. coli*, by the action of a fatty acid methyltransferase that uses *S*-adenosylmethionine as the donor for the methyl group [25]. However, the yield in this study was very low at roughly 15 μM FAME . Alternative products in the class of FAAE are wax esters, which are formed naturally by some plant species. Microbial production of wax esters in *E. coli* was first achieved by Kalscheuer and coworkers [226], who used the acyl-CoA reductase from the jojoba plant to reduce fatty acyl-CoA to fatty alcohol. The latter was esterified with fatty acyl-CoA, yielding wax esters. In this study, the maximal concentration of wax esters reached 1% of the cell dry weight. Steen *et al*. [26] demonstrated wax ester formation in *E. coli* Δ*fadE* upon overexpression of the genes for an acyl-CoA reductase (from *Mus musculus*), *tesA*, *fadD* and *atfA*. More recently wax ester synthesis was also established in the cyanobacterium *S. elongatus* by the overexpression of *atfA*, an acyl-ACP reductase and alcohol dehydrogenase [227].

The production of large amounts of fatty acid ethyl esters in microorganisms other than *E. coli* is so far restricted to *S. cerevisiae*[221,228], which is known to naturally synthesize small amounts of FAEE with medium chain-length [229]. FAEE production could potentially benefit from the endogenous ethanol production and tolerance of high ethanol concentrations of *S. cerevisiae*. However, the study of Yu *et al*. [228] shows that the endogenous ethanol production has to be boosted in order to achieve higher amounts of FAEE. The best result in this study was a concentration of 0.52 g l^-1^ after 72 hours of batch cultivation.

## Production of other fatty acid-derived biofuels

**Figure 4:**
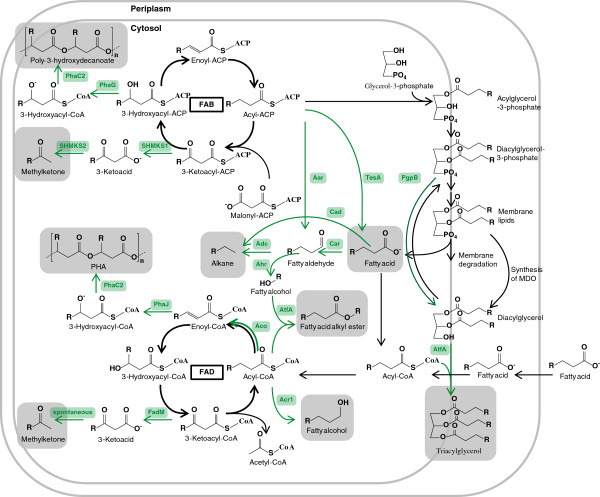
**Metabolic pathways for the production of fatty acids and derived compounds.** Enzymes and arrows are shown in green, if the pathway to which they belong departs from the wild-type fatty acid metabolism. The end products are shown in gray boxes. Appropriate deletions or homologous overexpressions within the wild-type pathway are not highlighted in this figure. For details, the reader is referred to the text. FAB, fatty acid biosynthesis; FAD, fatty -acid degradation; Aar, acyl-ACP reductase; Aco, acyl-CoA oxidase; Acr1, acyl-CoA reductase; Adc, aldehyde decarbonylase; Ahr, aldehyde recuctase; AtfA, acyltransferase; Cad, carboxylic acid decarboxylase; Car, carboxylic acid reductase; FadM, thioesterase; PgpB, phosphatidylglycerol phosphate phosphatase; PhaC2, PHA synthase 2; PhaG, (*R*)-3-hydroxydecanoyl-ACP transacylase; PhaJ, (*R*)-specific enoyl-CoA hydratase; SHMKS1, methylketone synthase 1; SHMKS2, methylketone synthase 2; TesA, thioesterase.

Besides free fatty acids and fatty acid alkyl esters some research has focused on the production of alternative biofuels that are derived from *E. coli* fatty acid biosynthesis. The native and engineered pathways of *E. coli* are summarized in Figure 4. Examples for this are triacylglycerols that also occur naturally in a variety of eukaryotic and prokaryotic cells, methyl ketones, alcohols and alkanes and polyhydroxyalkanoates with a medium chain-length.

### TAG

The formation of TAG is native to only a few bacterial genera, such as *Rhodococcus*, *Mycobacterium*, *Streptomyces*, *Nocardia*, *Acinetobacter* or *Alcanivorax*[230]. Of these the organisms *Rhodococcus opacus*[38,231-234], *Streptomyces coelicolor*[235-237] and species of the genus *Mycobacterium*[238-240] are the best studied. *R. opacus* has been shown to accumulate TAG up to 86% of the cellular dry weight [231] and was subject to pilot-scale fermentation and optimization [241-243].

Attempts to establish TAG formation in *E. coli* have been very rare. In 2008 Arabolaza and coworkers [235] transformed an *E. coli dgkA* (diacylglycerol kinase A) mutant with plasmids containing three different WS/DGAT enzymes from *S. coelicolor*. The *dgkA* mutant of *E. coli* has earlier been found to accumulate high levels of diacylglycerol, due to an impaired membrane lipid-recycling following to the synthesis of membrane-derived oligosaccharides [111,244,245]. Expression of one of the investigated WS/DGAT enzymes (Sco0958) led to TAG formation in the mutant strain [235].

An alternative way has been investigated more recently [236,246], which employs enzymes that catalyze the dephosphorylation of phosphatidic acid, yielding diacylglycerol. The first study used the overexpression of the *atfA* and *E. coli pgpB* (phosphatidate phosphatase) genes, by which the synthesis of 1.1 mg l^-1^ TAG was achieved. The study of Comba *et al*. [236] used the same biosynthetic route, but with the genes *Sco0958* and *lppα* or *lppβ* (phosphatidate phosphatases) from *S. coelicolor*.

Apart from *E. coli*, heterologous TAG formation has also been performed in the cyanobacterium *S. elongatus*, by overexpression of *atfA*[227]. As *S. elongatus* uses an acyl-ACP synthetase instead of an acyl-CoA ligase for fatty acid activation [247], this raises the question as to whether AtfA can also use acyl-ACP as substrate. Optimization of the native TAG production in algae by metabolic engineering also offers great potential for the production of next-generation biofuels [248,249].

### Methyl ketones

Methyl ketones are formed by the hydrolysis of an acyl-ACP intermediate and the subsequent decarboxylation of the 3-keto acid. These volatile substances were first found in rue (*Ruta graveolens*) [250] but are widespread among plant, animal and microbial species [251]. Wild-type *E. coli* cells do not produce significant amounts of methyl ketones, but the ability can be established by metabolic engineering. In the first study small amounts of methyl ketones were obtained by overexpression of the genes *shmks1* and *shmks2* (methylketone synthases 1 and 2) from wild tomato (*Solanum habrochaites*) [252]. Park *et al*. [253] applied overexpression of these genes in an *E. coli* strain that was blocked in four pathways of the fermentation metabolism by deletion of the genes *adhE*, *ldhA*, *poxB* and *pta*. This strain procuced 450 mg l^-1^ methyl ketones. Shortly before, a methyl ketone titer of 380 mg l^-1^ was published upon overexpression of the genes *fadB*, *fadM* and *Mlut11700* (an acyl-CoA oxidase of *Micrococcus luteus*) in an *E. coli* strain with deleted *fadE* and *fadA* genes [254]. The combination of the genes *fadB*, *fadM* and *Mlut11700* was also sufficient for chemolithoautotrophic production of up to 180 mg l^-1^ methyl ketones in a strain of *Ralstonia eutropha* with both *β*-oxidation operons deleted [255].

### Fatty alcohols and alkanes

Another way to process fatty acids for fuel production is the reduction to long-chain alcohols, alkanes and alkenes. First attempts have been performed by Keasling and coworkers [256], who showed that an *E. coli fadE* mutant produced small amounts of fatty alcohols. Additional overexpression of *fadD*, *acr1* (acyl-CoA reductase 1 from *A. calcoaceticus* BD413) and *tesA* enhanced the fatty alcohol production to 60 mg l^-1^[26]. Another study has shown that the overexpression of only a fatty acyl-CoA reductase (in this case from *Arabidopsis thaliana*) is sufficient for fatty alcohol production in otherwise unmodified wild-type *E. coli*[257]. By reversal of the *β*-oxidation pathway [201] (see Section ‘Altering the regulation or pathway direction’ for details) and overexpression of an alcohol dehydrogenase, 330 mg l^-1^ n-alcohols with chain lengths of 5 to 10 carbon atoms were produced. A slightly higher concentration of 350 mg l^-1^ fatty alcohols was achieved by expression of the carboxylic acid reductase (*Mycobacterium marinum*), *ahr* (aldehyde reductase of *E. coli*) and *tesA* genes [258].

The biosynthesis of alkanes and alkenes is not done by further reduction of a fatty alcohol, but by a decarboxylation or decarbonylation of a fatty acid or aldehyde. In 2010 Lennen *et al*. reported on the production of alkanes by the conversion of fatty acids, extracted from an overproducing strain of *E. coli*[202]. Complete biosynthesis of alkanes was achieved by overexpression of acyl-ACP reductase and aldehyde decarbonylase (both from *S. elongatus*) in *E. coli*[259]. As these enzymes can use acyl-ACP, the coexpression of *fadD* does not lead to higher productivity. Presumably, a thioesterase overexpression would also rather diminish fatty alkane production. By coexpression of *fabH2* (*β*-ketoacyl-ACP synthase of *B. subtilis*) up to 80 mg l^-1^ alkanes with even and uneven chain length could be produced [260].

A similar system based on FFA took advantage of the genes *luxCED* (fatty acid reductase complex from *Photorhabdus luminescens*) and *NpAD* (aldehyde decarbonylase from *Nostoc punctiforme*) for the production of fatty alkanes [261]. By coexpression of *fabH2*, branched-chain fatty acids were also produced and processed to the respective alkanes. The coexpression of *fatB* (thioesterase of *C. camphora*, specific for tetradecanoyl-ACP) resulted mainly in the synthesis of tridecane. However, the yields of the alkane production were lower than 10 mg l^-1^, also with *fatB* overexpression [261]. In the study of Akhtar and coworkers [258] a carboxylic acid reductase of *M. marinum* and an aldehyde decarboxylase of *Prochlorococcus marinus* were used in combination with *tesA* expression. The exact yields were not given, but it was stated that the yields were considerably lower than for fatty acids or alcohols.

The production of 1-alkenes by the decarboxylation of FFA has been studied in the Gram-positive bacterium *Jeotgalicoccus* sp. ATCC 8456. Identification of the responsible gene (a fatty acid decarboxylase) revealed that heterologous expression is sufficient for one-step production of 1-alkenes in *E. coli*[262].

### PHA_mcl_

Polyhydroxyalkanoates (PHA) are polymers that can be used as biodegradable plastics. However, their physical properties and thus their usability depend on the kind of the monomer (s) [263]. Whereas the well-investigated and first-discovered polyhydroxybutyrate (PHB) is synthesized by the condensation of two molecules of acetyl-CoA (reaction of PhaA) and the subsequent reduction of acetaldehyde (by PhaB) and polymerization of 3-hydroxybutyrate (by PhaC) [264], the monomers of PHA with longer carbon chains typically are taken from fatty acid biosynthesis [265] or degradation [266]. Biosynthesis of these PHA_mcl_ (with medium chain-length) also occurs in many prokaryotes and is well studied in species of the genus *Pseudomonas*[267] that were also genetically modified [268-270]. The monomers of PHA with medium or long alkyl chains are 3-hydroxy fatty acids and in addition to their use as bioplastics may be considered as potential biofuels.

Aiming to establish the production of PHA_mcl_ in *E. coli*, the cells are made to overproduce or grow on FFA that enter the *β*-oxidation, and the intermediate product 3-hydroxyacyl-CoA is then polymerized by a suitable PhaC (PHA synthase) enzyme. In the first studies concerning PHA_mcl_ production in *E. coli*, this was achieved by expression of the PHA synthases 1 and 2 of *Pseudomonas aeruginosa* in *E. coli* mutants, impaired in the *β*-oxidation and with acrylic acid as the inhibitor [271,272].

Klinke *et al*. [273] reported the synthesis of PHA_mcl_ up to 2.3% of the cell dry weight by overexpression of *tesA* and *phaC*_*mcl*_ from *P. aeruginosa*. With the same PHA polymerase and the thioesterase of *U. californica*, coexpressed in an *E. coli fadB* mutant 6% of the cell dry weight could be achieved [274]. The effect of *fadA*, *fadB* or *fadAB* mutant strains was further studied by Park and Lee [275]. By the coexpression of *phaA*, *phaB* (both of *R. eutropha*) and *phaC2* and with decanoate feeding, the authors showed the production of PHB-PHA_mcl_ copolymers with different ratios of PHB to PHA_mcl_, depending on the deletion mutant used.

A step towards the production of PHA homopolymers with medium chain-length-3-hydroxyalkanoates as constituents has been the production of 3-hydroxydecanoate, up to 46% of the cell dry weight by the combined expression of *tesB* (thioesterase II from *E. coli*) and *phaG* ((*R*)-3-hydroxydecanoyl-ACP transacylase from *P. putida*) [276]. However, a polymerization of 3-hydroxydecanoate has not been reported in this study. Further attempts towards the production of copolymers with short and medium chain-lengths have been the coexpression of several mutant genes of *fabH* and *phaC1* (from *Pseudomonas* sp. 61–3) with *phaA* and *phaB* (from *R. eutropha*) [277] and additionally with *fabG*[82]. These studies succeeded in the production of PHA with an enhanced proportion of monomers with 6, 8, 10 and 12 carbon atoms. By application of an *E. coli fadR* and *atoC* mutant, the polymer content could be further enhanced [83]. To date, the highest amount of PHA copolymer (15% of the cell dry weight) from non-related carbon sources has been achieved by Agnew *et al*. [278]. They expressed the *tesA* gene for FFA production in combination with the *P. aeruginosa* genes *phaC*, *phaJ* ((*R*)-specific enoyl-CoA hydratase) and PP_0763 (putative acyl-CoA synthetase), in an *E. coli* strain with deletions in *fadR*, *fadI*, *fadJ* (also named *yfcX*), *fadA* and *fadB*.

## Feedstocks

If one wishes to produce microbial biofuels (or bulk chemicals) in a cost-competitive way with petrochemical or oil plant-based production processes, there are two key factors to consider. First, it is important to achieve high product-concentrations, as that will influence the productivity of a fermentation plant and maximize the yields in product recovery and refinement. The second key factor is the cost for the carbon source [279]. In far-developed processes, the carbon source can account for 30 to 60% of the production costs, as is the case for the production of polyhydroxybutyrate from glucose [280], propanediol from glycerol [281] or ethanol from sugar cane molasses [282].

Glucose is the most common substrate for bacterial growth and is used in most of the studies that were performed in the field of biofuel production with engineered *E. coli*. However, glucose is rather expensive, whereas sucrose from sugar cane was described as the cheapest carbon source available for industrial fermentations in 2004 [283]. Also for future development, sucrose was expected to be at least as competitive (on price) as lignocellulosic biomass for the carbon source [283].

Utilization of sucrose is limited to a few *E. coli* strains, but can be established in *E. coli* K-12 by overexpression of *cscA* and *cscB*, coding for the invertase and the sucrose transporter [284]. However, as mentioned in the introduction, biofuel synthesis based on sucrose utilization will lead to competition with food production and to the use of arable land. Thus, the use of lignocellulosic biomass would be promising, but it typically includes severe pretreatment of the material, to break down the dense cellulose fibers [285]. Currently, this pretreatment involves acid- or base-catalyzed hydrolysis and the addition of cellulase enzymes, and hence is quite expensive. Furthermore, a sideproduct of this pretreatment is the formation of furfural, which inhibts bacterial growth. This problem has been addressed in a recent publication, and by expression of *fucO*, *ucpA* and *pntAB* and deletion of *yqhD*, the furfural tolerance of *E. coli* could be enhanced [286]. Another problem is that lignocellulosic biomass (once degraded to its components) consists of several different sugars that are utilized sequentially, due to the catabolite repression system of *E. coli*. This sequential degradation leads to many short *lag* phases, when the bacteria switch from one consumed carbon source to another. To circumvent this problem, regulatory mutant strains can be used [287]. However, degradation of the phenolic compounds from the lignin moiety of lignocellulose is still not possible for *E. coli* strains and reduces the possible product yield on this substrate. Besides lignocellulose, cheap substrates that can be utilized by *E. coli* include cheese whey [288,289] or seaweed hydrolysate [290].

Most studies have addressed either the production of biofuels or the growth of *E. coli* on alternative carbon sources. However, direct production of FAEE has been reported using hemicellulose sugars [26] and pretreated switchgrass [20,225].

## Conclusions

Of the products that were discussed in this review, free fatty acids and PHAs from genetically engineered *E. coli* were the subject of most studies and have consequentely yielded the best results. The production of FAEE has only yielded high concentrations if an even higher amount of oleic acid has been supplemented to the medium (in addition to the primary carbon source) [222]. Methyl ketones, fatty alcohols and alkanes are being synthesized only with low yields, but research on their microbial production has just begun. Triacylglycerols can already be produced naturally by many microbes. Engineering of *E. coli* towards TAG production will only lead to a competetive process if it is possible to combine a high TAG-accumulation with the fast growth of *E. coli*.

Concerning the productivity of fatty acid synthesis it is difficult to estimate the current state of the art, since many studies have been performed only in batch mode. The theoretical limit of fatty acid production with glucose as the carbon source is roughly 35% (w/w). With some engineered strains, 56 to 85% of the theoretical limit has been reached [201,291] by metabolic engineering and by modifications in the regulation of fatty acid metabolism. However, the highest-reported product concentrations approached 10 g l^-1^, with productivities in the range of 0.1 to 0.2 g l^-1^ h^-1^[106,142,143,201,292]. These values indicate that much research still needs to be done to reduce the time for the production of high concentrations of fatty acids. As cell growth and fatty acid production compete for the carbon source, it will be necessary to develop a continuous fermentation or a repeated fed-batch process with high cell-densities that enable high product-concentrations. A problem may then arise, because free fatty acids can impose a considerable stress on the cells if present in high concentrations. Thus, a continuous extraction or the conversion to non-toxic endproducts seems to offer great potential for future processes and strain improvements. PHA or TAG are such end products, but they accumulate in the cells, which makes recycling of living cells impossible. FAEE might be suitable and have been shown not to be growth-inhibitory up to concentrations of 100 g l^-1^[26]. Finally it is desireable to combine biofuel production with the engineered ability to grow on cheap resources like cellulose or hemicellulose. First attempts have already been made to show the general possibility [26,225].

## Abbreviations

Acc: Acetyl-CoA carboxylase; ACP: Acyl carrier protein; BC-BCCP: Biotin carboxylase-biotin carboxyl carrier protein; CRP-cAMP: Cyclic adenosine monophosphate receptor protein-cyclic adenosine monophosphate complex; FAAE: Fatty acid alkyl ester; FAEE: Fatty acid ethyl ester; FFA: Free fatty acid; (p)ppGpp: (guanosine 5-triphosphate, 3-diphosphate) guanosine 3,5-bispyrophosphate; PHA: Polyhydroxyalkanoate; PHB: Polyhydroxybutyrate; TAG: Triacylglycerol; tesA: Leaderless version of the gene for the *E. coli* thioesterase I, that remains in the cytosol; WS/DGAT: Wax ester synthase/acyl-CoA: Diacylglycerol acetyl transferase.

## Competing interests

The authors declare that they have no competing interests.

## Authors’ contributions

HJ conceptualized, researched and wrote the manuscript. AS conceptualized, helped to draft the manuscript and critically revised it. Both authors read and approved the final manuscript.
